# Swi1^Timeless^ Prevents Repeat Instability at Fission Yeast Telomeres

**DOI:** 10.1371/journal.pgen.1005943

**Published:** 2016-03-18

**Authors:** Mariana C. Gadaleta, Mukund M. Das, Hideki Tanizawa, Ya-Ting Chang, Ken-ichi Noma, Toru M. Nakamura, Eishi Noguchi

**Affiliations:** 1 Department of Biochemistry and Molecular Biology, Drexel University College of Medicine, Philadelphia, Pennsylvania, United States of America; 2 Gene Expression and Regulation Program, The Wistar Institute, Philadelphia, Pennsylvania, United States of America; 3 Department of Biochemistry and Molecular Genetics, College of Medicine, University of Illinois at Chicago, Chicago, Illinois, United States of America; National Institute of Biological Sciences, CHINA

## Abstract

Genomic instability associated with DNA replication stress is linked to cancer and genetic pathologies in humans. If not properly regulated, replication stress, such as fork stalling and collapse, can be induced at natural replication impediments present throughout the genome. The fork protection complex (FPC) is thought to play a critical role in stabilizing stalled replication forks at several known replication barriers including eukaryotic rDNA genes and the fission yeast mating-type locus. However, little is known about the role of the FPC at other natural impediments including telomeres. Telomeres are considered to be difficult to replicate due to the presence of repetitive GT-rich sequences and telomere-binding proteins. However, the regulatory mechanism that ensures telomere replication is not fully understood. Here, we report the role of the fission yeast Swi1^Timeless^, a subunit of the FPC, in telomere replication. Loss of Swi1 causes telomere shortening in a telomerase-independent manner. Our epistasis analyses suggest that heterochromatin and telomere-binding proteins are not major impediments for telomere replication in the absence of Swi1. Instead, repetitive DNA sequences impair telomere integrity in *swi1*Δ mutant cells, leading to the loss of repeat DNA. In the absence of Swi1, telomere shortening is accompanied with an increased recruitment of Rad52 recombinase and more frequent amplification of telomere/subtelomeres, reminiscent of tumor cells that utilize the alternative lengthening of telomeres pathway (ALT) to maintain telomeres. These results suggest that Swi1 ensures telomere replication by suppressing recombination and repeat instability at telomeres. Our studies may also be relevant in understanding the potential role of Swi1^Timeless^ in regulation of telomere stability in cancer cells.

## Author Summary

In every round of the cell cycle, cells must accurately replicate their full genetic information. This process is highly regulated, as defects during DNA replication cause genomic instability, leading to various genetic disorders including cancers. To thwart these problems, cells carry an array of complex mechanisms to deal with various obstacles found across the genome that can hamper DNA replication and cause DNA damage. Understanding how these mechanisms are regulated and orchestrated is of paramount importance in the field. In this report, we describe how Swi1, a Timeless-related protein in fission yeast, regulates efficient replication of telomeres, which are considered to be difficult to replicate due to the presence of repetitive DNA and telomere-binding proteins. We show that Swi1 prevents telomere damage and maintains telomere length by protecting integrity of telomeric repeats. Swi1-mediated telomere maintenance is independent of telomerase activity, and loss of Swi1 causes hyper-activation of recombination-based telomere maintenance, which generates heterogeneous telomeres. Similar telomerase-independent and recombination-dependent mechanism is utilized by approximately 15% of human cancers, linking telomere replication defects with cancer development. Thus, our study may be relevant in understanding the role of telomere replication defects in the development of cancers in humans.

## Introduction

Eukaryotic cells must accurately replicate their genetic information every cell cycle. However, this process is challenged by the presence of natural impediments throughout the genome that can halt replisome progression, potentially causing genomic instability, a hallmark of cancer and other hereditary disorders [1–4]. These natural impediments are termed replication fork barriers (RFBs) and are typically classified into two groups. The first group arises from non-histone DNA-binding proteins, such as fork-blocking proteins found at eukaryotic rDNA genes, the fission yeast mating-type locus, and long terminal repeats (LTRs) [5–9]. The second group includes DNA secondary structures such as G quadruplexes, hairpins, and triplex DNA, which are often found at repetitive or palindromic DNA sequences [10–15]. Although these RFBs present obstacles for DNA replication, the nature of these barriers and the mechanisms by which the cell ensures the smooth passage of the replisome through each RFB are not fully understood.

Key to the replisome regulation at RFBs is the fork protection complex (FPC), which is composed of the Timeless and Tipin proteins in mammalian cells. The functions of the FPC are conserved throughout evolution, reflecting its fundamental role in genome stability [16, 17]. The FPC travels with the replisome during DNA replication, stabilizes stalled forks, and promotes full activation of the DNA replication checkpoint [18–25]. The FPC also plays a critical role at several RFBs. In the fission yeast *Schizosaccharomyces pombe*, Swi1 (Timeless orthologue) and Swi3 (Tipin orthologue) are necessary for programmed fork termination and pausing at *RTS1* (replication termination site 1) and *MPS1* (*mat1* pausing site 1) at the mating-type locus (*mat1*), respectively, leading to an imprinting event required for mating-type switching [5, 26, 27]. Swi1 and Swi3 are also required for site-specific replication fork arrest at the Ter1-3 sites within the intergenic spacer of the rDNA repeats [28]. Furthermore, Swi1 prevents recombinogenic lesions at RFBs localized at tRNA genes in fission yeast [29].

In addition to the RFBs described above, studies in yeast and human cells have shown that telomeres form specialized chromatin structures that cause replication fork pausing during telomere replication [30–32]. Telomeres are nucleoprotein structures at the ends of linear chromosomes in eukaryotes. Repetitive G-rich sequences at telomeres can form G-quadruplex structures that may hinder DNA replication. Telomeres also recruit heterochromatin proteins and telomere-specific proteins that form the shelterin complex [33–35]. Such structures may cause genomic instability during DNA replication if this process is not properly regulated.

In fission yeast, the RNA interference (RNAi) pathway initiates heterochromatin formation, leading to Clr4-dependent histone H3 methylation and subsequent recruitment of Swi6, the orthologue of heterochromatin protein 1 (HP1) [36, 37]. At telomeres, heterochromatin establishment can occur by an additional pathway that uses Taz1, a component of the shelterin complex, which regulates telomerase activity and inhibits DNA damage response at telomeres [38]. Taz1 contains a Myb DNA-binding domain, associates with telomeric repeats, and plays a role in efficient replication of telomeres [39]. In mammalian cells, deletion of TRF1, a Taz1 orthologue, results in inefficient telomere replication when monitored by DNA combing, while overexpression of TRF1 can also lead to fork stalling at telomere repeats [40, 41]. These results suggest that the shelterin complexes including Taz1/TRF1 play a critical role in regulation of telomere replication. Previously, we have found that Timeless (Swi1 orthologue) interacts with TRF1, a component of shelterin, and prevents telomere shortening and fork collapse at telomeres in human cells [42]. In *S*. *cerevisiae*, loss of Tof1 (Swi1 orthologue) or its partner Csm3 (Swi3 orthologue) causes an increase in telomere-size heterogeneity but not significant telomere shortening [43]. These findings suggested a role of the FPC in telomere replication. However, whether the function of the FPC at telomere is conserved throughout evolution is not known, nor is it known what features of telomeres actually present the barrier to the DNA replication process.

In this study, we investigated the role of Swi1 in fission yeast telomere maintenance. We show that Swi1 deletion results in telomere shortening, accumulation of DNA damage, and increased recombination at telomeres. Our results suggest that the primary obstacle for unstable replisomes lacking Swi1 may lie in the repetitive nature of the telomeric DNA and not in the presence of heterochromatin or non-histone DNA-binding proteins. We also show that the telomere shortening in *swi1Δ* cells is likely caused by replication problems and not by defects in telomerase recruitment. Furthermore, we show that *swi1*Δ cells more frequently utilize alternative lengthening of telomeres (ALT)-like mechanisms than wild-type cells to amplify telomeric/subtelomeric regions in the absence of telomerase to maintain telomeres. Taken together, our analyses reveal novel and conserved roles of the FPC in telomere maintenance and in replication fork stabilization at repetitive DNA regions. Considering that approximately 10–15% of all human cancers activate ALT pathways to maintain telomeres [44, 45] and that ALT was recently shown to occur even in normal mammalian somatic cells *in vivo* [46], our results may be relevant in understanding how Swi1^Timeless^ ensures telomere stability and prevents ALT activation in subsets of human cancers.

## Results

### Swi1 prevents DNA damage at telomeres

We previously found that *swi1*Δ cells accumulate spontaneous Rad52 DNA repair foci during DNA replication [47]. Rad52 recombinase binds single-stranded DNAs (ssDNAs) at sites of DNA damage and is required for DNA repair [48, 49], indicating that *swi1*Δ cells accumulate DNA lesions. However, it was unknown whether these Rad52 foci are localized randomly throughout the genome or at specific chromosome loci in *swi1*Δ cells. Since human Timeless, the ortholog of *S*. *pombe* Swi1, is involved in preventing telomere damage [42], we hypothesized that Rad52 is preferentially recruited to telomeres in *swi1*Δ cells. To address this hypothesis, we performed ChIP-seq analysis of Rad52 using wild-type and *swi1*Δ cells endogenously expressing Rad52 fused to 12 tandem copies of the Pk epitope (Rad52-12Pk). We previously reported that Rad52-12Pk is functional as *rad52-12Pk* cells showed no significant change in DNA damage sensitivity, and Rad52-12Pk was recruited to the *mat1* locus in wild-type cells [50].

**Fig 1:**
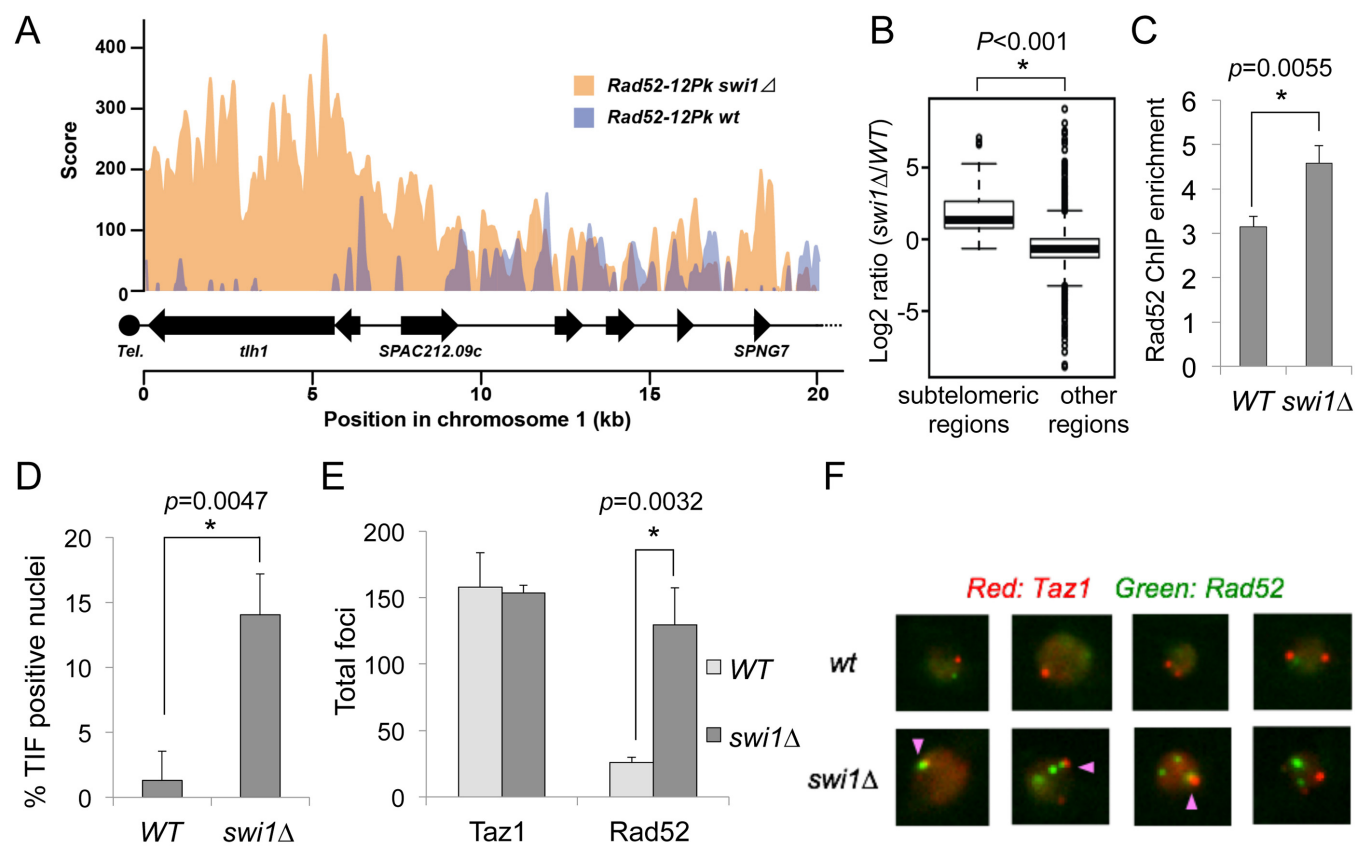
Swi1 loss causes DNA damage at telomeres. **(A)** Genome-wide (ChIP-seq) analysis of Rad52 distribution at the subtelomeres (left arm of chromosome 1) in asynchronous cultures of wild-type (green) and *swi1Δ* (blue) cells. Enrichment of Rad52 is displayed as enrichment scores (y-axis). Chromosome coordinates [x-axis, in megabases, (Mb)] were downloaded from the *S*. *pombe* Genome Project (Sanger Center: www.sanger.ac.uk/Projects/S_pombe). The strains used for our experiments are both heterothallic *h*^*+*^ strains. **(B)** Box plots for log2 ratios of Rad52 enrichment at subtelomeric regions vs other regions. The boxes were limited by the 25th and 75th percentiles, with the black lines representing the median ratios between *swi1*Δ and wild-type. The whiskers are extended out to the most extreme data points that are at most 1.5 times the interquartile range from the box. The black circles represent outliers. The *p*-value was evaluated by Mann-Whitney U test. **(C)** ChIP assays showing Rad52 enrichment at telomeres in wild-type and *swi1*Δ cells. Rad52-12Pk was immunoprecipitated from the indicated cells, and associated DNA was subjected to competitive multiplex PCR to amplify DNA fragments from the *TAS1* region and a gene-free region (GFR2), which was used as an internal amplification control. Chromatin association of Rad52-12Pk at *TAS1* was presented as relative enrichment over the association at GFR2. Data are expressed as the mean of three independent experiments. Error bars represent the standard deviation. *p*-value was determined by two-tailed Student's t-test. **(D-E-F)** Wild-type and *swi1Δ* cells expressing Rad52-YFP and Taz1-mCherry were grown in minimal medium at 25°C until mid-log phase. Nuclei (n = 102) were analyzed for the percentage of TIF-positive nuclei in (D) as well as for the total number of Rad52-YFP and Taz1-mCherry foci in (E). Accumulation of Rad52-YFP foci (E) and a significant increase in TIF-positive nuclei were observed in the *swi1Δ* cells (D). Data shown is the mean of three independent experiments. Error bars represent the standard deviation. *p*-values were determined by two-tailed Student's t-test. (F) Representative microscopic images. Pink arrows indicate TIFs.

Interestingly, Rad52-12Pk ChIP-seq analysis showed that subtelomeric regions have increased Rad52 binding in *swi1*Δ cells compared to wild-type cells (Fig 1A). In order to confirm the specific enrichment of Rad52 at subtelomeric regions, we compared Rad52 enrichment between subtelomeres and other chromosome regions (Fig 1B). For this purpose, we divided the entire genome into non-overlapping 2-kb windows and compared Rad52 enrichment at each 2-kb window between *swi1*Δ and wild-type samples. We accounted 20-kb from each chromosome end as a subtelomere region (ten 2-kb windows from a chromosome end). We excluded rDNA regions from the analysis because Rad52 is enriched at rDNAs in *swi1*Δ cells (described later). The box plots for log2 ratios of Rad52 enrichment showed a significant increase of Rad52 association at subtelomeres over other chromosome regions in *swi1*Δ cells (Fig 1B).

**Fig 2:**
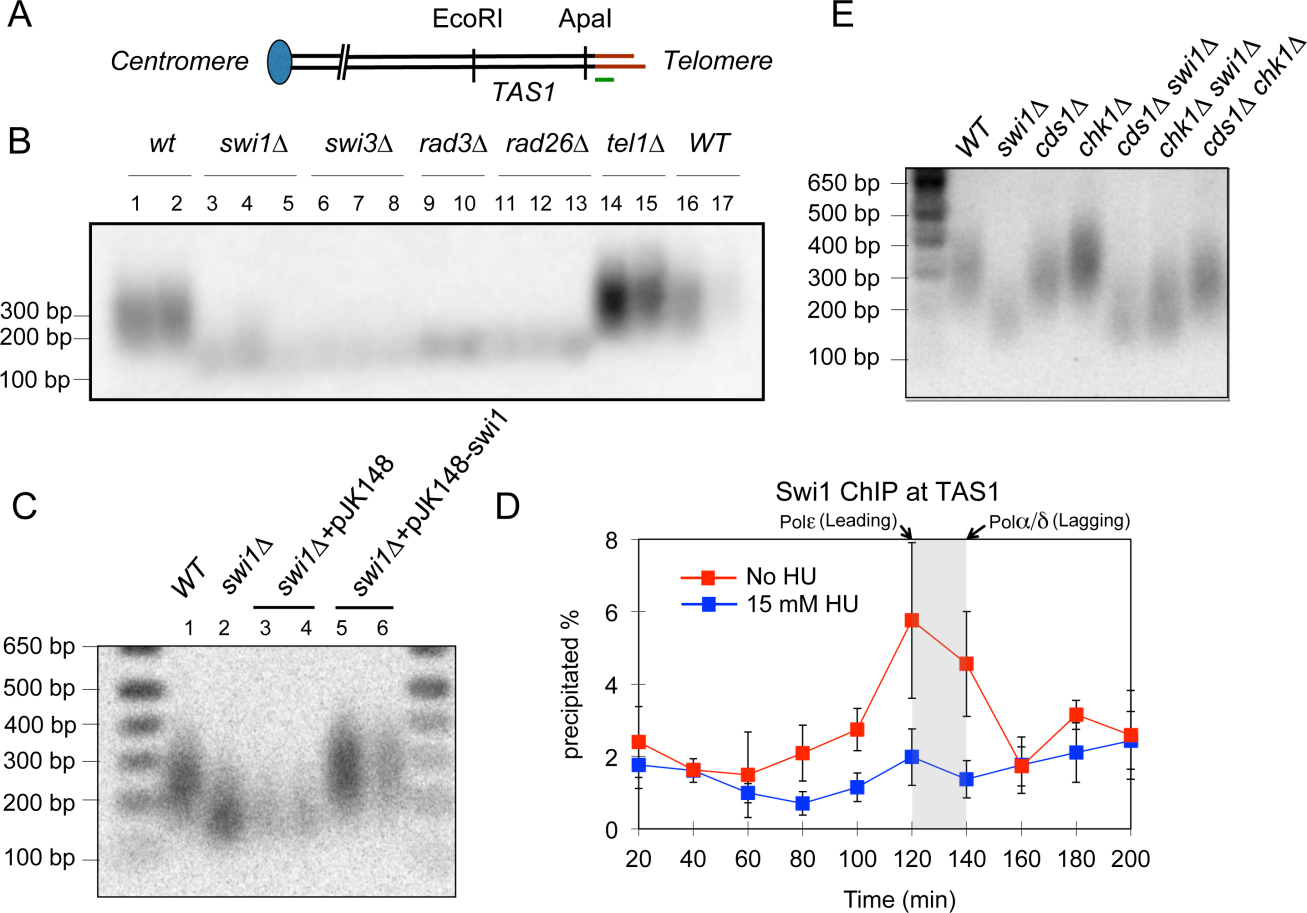
The Swi1-Swi3 FPC is required for telomere maintenance. **(A)** Schematic diagram showing the position of the *Apa*I restriction site at *S*. *pombe* telomeres and the *TAS1* (Telomere-Associated Sequences) region [87]. Terminal telomeric repeats are shown in red. The position of the telomeric probe used in this study is marked in green. **(B)** Southern blot analysis of *Apa*I-digested genomic DNA from independently isolated strains of the indicated genotypes. Each strain was passaged at least 8 times before DNA isolation, in order to allow for telomere length stabilization. *Apa*I-telomere fragments were detected with a telomere-specific DNA probe. **(C)** *swi1*Δ cells were transformed with the pJK148-Swi1 plasmid or with the control vector pJK148. Transformants were isolated and passaged at least 8 times before genomic DNA preparation. Southern blot analysis of the *Apa*I-telomere fragments was performed as described above. Telomeres of two or three independent isolates from each transformation are shown. **(D)** Swi1 is recruited to *TAS1* in a replication-dependent manner. *swi1-13Myc cdc25-22* cells were synchronized at the G2/M boundary by incubation at 36°C for 3 h and then released into fresh YES medium with or without 15 mM hydroxyurea (HU), an inhibitor of DNA replication. Cells were collected at the indicated times and processed for Swi1-13Myc ChIP, and telomere association of Swi1 was determined by real-time PCR as described [127]. Swi1-13Myc was associated with *TAS1* as cells replicate DNA. Previously determined peaks of binding for leading (Pol ε) and lagging (Pol α and Pol δ) strand DNA polymerase [52] are indicated. Addition of HU, which prevented telomere replication, inhibited Swi1-13Myc recruitment at *TAS1*. **(E)** Southern blot analysis of *Apa*I-telomere fragments from the indicated cells was performed as described in *B*.

To address the role of Swi1 in telomere maintenance, we also performed PCR-based ChIP analysis of Rad52 at telomeres. We found that in wild-type cells, Rad52 enrichment at subtelomeres (the *TAS1* region) was approximately 3 times more than that at a control locus (Fig 1C, see Fig 2A for location of TAS1 subtelomeric region), indicating that telomeres are inherently difficult to replicate and are prone to generating ssDNA. These results are in agreement with previous reports showing differential arrival of the leading- and lagging-strand DNA polymerases and an S phase-specific increase in RPA recruitment at telomeres [51, 52]. Importantly, Rad52 binding at the *TAS1* region was further increased in *swi1*Δ cell (Fig 1C), suggesting the role of Swi1 in limiting Rad52 accumulation at telomeric regions. To further substantiate this result, we conducted a telomere-dysfunction induced foci (TIF) assay that examines co-localization of Rad52 and a telomere maker, Taz1. For this purpose, Rad52 and Taz1 were fused to yellow fluorescent protein (YFP) and mCherry, respectively, and expressed from their own promoters at their chromosomal loci. We counted over 100 nuclei and determined the ratio of TIF-positive nuclei in each cell culture. This analysis revealed that *swi1*Δ cells present a higher amount of TIF-positive nuclei than wild-type cells (Fig 1D and 1F). *swi1Δ* cells did not show any significant difference in the number of Taz1 foci; however, they displayed more Rad52 foci overall than wild-type cells (Fig 1E and 1F). Therefore, it was possible that the elevated TIF-positive nuclei seen in the *swi1*Δ culture was a consequence of increased stochastic DNA damage events occurring throughout the genome. To exclude this possibility, we next calculated the ratio of TIF events to total Rad52 foci. TIF occurred in 5.19% and 11.08% of Rad52 foci in wild-type and *swi1*Δ cells, respectively (*see* S1A Fig), indicating that telomeres undergo increased DNA damage in the absence of Swi1. In addition, we observed the occurrence of Rad52 foci lateral to Taz1 foci in *swi1*Δ cells (S1B Fig). This may reflect the Rad52 enrichment found at subtelomeres in our Rad52 ChIP-seq experiments (Fig 1A), and subtelomeric regions are also susceptible to DNA damage. However, these Rad52 foci lateral to Taz1 foci were not considered as TIFs in our analysis. Thus, increase in DNA damage occurring at telomeric/subtelomeric regions is likely to be even more severe than suggested by our analysis of TIF-positive cells. Taken together, our results established the critical role of Swi1 in preventing DNA damage at telomeres in fission yeast.

### Swi1 is required for telomere length maintenance

Although the role of the FPC in telomere maintenance has been suggested [16, 42, 53], mechanistically how Swi1 regulates telomere length is unknown. In order to understand the role of the FPC in telomere replication, we first investigated the role of the Swi1-Swi3 FPC in telomere length maintenance. Cells were consecutively streaked at least 8 times on YES plates at 3-day intervals (~33 doublings per streak) in order to stabilize telomere lengths after deletion mutants were generated. To detect telomere fragments, the genomic DNA from several independent isolates of FPC mutants was digested with *Apa*I (Fig 2A) and analyzed by Southern blot (Fig 2B). Consistent with previous reports [42, 53], *swi1*Δ cells had much shorter telomeres when compared to wild-type cells (Fig 2B). In addition, we found that *swi3*Δ cells carry short telomeres similar to *swi1*Δ (Fig 2B). Telomere lengths in *swi1Δ* and *swi3Δ* cells were similar to those in *rad3Δ* and *rad26Δ* mutants, already known to harbor significantly short telomeres [54–56] (Fig 2B). Telomere shortening in *swi1Δ* cells was rescued by transforming *swi1Δ* cells with a plasmid vector containing the *swi1*^+^ gene but not with a control vector (Fig 2C), indicating that the loss of telomeric repeats is directly caused by loss of Swi1 and can be reversed by reintroducing a fully functional FPC. Furthermore, a cell cycle-ChIP analysis found that Swi1 is specifically recruited at telomeres during S phase and that addition of HU, which have been previously established to inhibit late-S phase replication of telomeres [57], eliminated Swi1 recruitment (Fig 2D). These observations suggested that the Swi1-Swi3 FPC is important for proper replication of telomeres to ensure telomere length maintenance in fission yeast.

The FPC stabilizes replication forks and mediates activation of the replication checkpoint [18, 21, 47]. In the latter function, Swi1 is required for proficient activation of the checkpoint kinase Cds1. However, previous reports indicated that Cds1 has no role in telomere length maintenance [54, 56, 58]. Consistently, we also observed that *cds1*Δ cells do not show telomere shortening (Fig 2E). In the absence of Swi1, cells accumulate replication stress, leading to Chk1-dependent G2/M delay, and *swi1*Δ *chk1*Δ cells shows growth defects and are much more sensitive to genotoxic agents than either single mutant [47]. Therefore, we tested whether telomere length in *swi1*Δ cells is affected by loss of Chk1. However, there was no further shortening of telomere length in *swi1*Δ *chk1*Δ cells compared to *swi1Δ* cells (Fig 2E). Thus, we concluded that Swi1-mediated telomere length maintenance does not rely on Swi1’s role in regulation of checkpoint kinases Chk1 and Cds1.

### Swi1 ensures replication of repetitive DNA

Several telomeric features can hamper the passage of the replication machinery and cause telomere damage and shortening. G-quadruplexes, repetitive DNA, heterochromatin, and the potential to form t-loop structures have been suggested as possible obstacles for the replication machinery; however the concrete nature of the telomere barrier remains elusive. We hypothesize that *swi1* deletion results in an unstable replisome that will be more vulnerable to these obstacles. Since previous studies have found that lagging-strand synthesis at fission yeast telomeres is substantially delayed compared to leading-strand synthesis [52, 59, 60], the FPC could be especially important for protecting integrity of replisome at telomeres until lagging-strand synthesis is successfully completed.

To determine whether Swi1 generally functions in maintaining integrity of repetitive DNA sequences, we first tested the role of Swi1 during replication in maintaining stability of *E*. *coli LacO* array derived from the pSV2-DHFR-8.32 vector [61]. This array contains 32 direct repeats of a 317-bp DNA fragment. Each fragment includes 8 direct repeats of a 36-bp DNA sequence containing the *LacO* operator and a 29-bp linker sequence; thus, this *LacO* array has 256 repeats of the 36-bp DNA sequence [61]. In order to investigate whether the *LacO* repeats interfere with the replication process, we used *S*. *pombe* strains that carry the *LacO* array at the *ade3*^*+*^ or *ade1*^*+*^ locus [62, 63] as these loci are not associated with known repeat DNA sequences [64, 65]. These strains were initially designed to express the LacI-GFP fusion protein that binds the *LacO* repeats inserted at *ade3* or *ade1* loci. Since LacI-GFP may affect replication of the *LacO* array [66], we removed the *LacI-GFP* transgene from these strains by genetic crossing before the *swi1*^*+*^ gene was deleted. Absence of *LacI-GFP* expression was confirmed by fluorescent microscopy.

**Fig 3:**
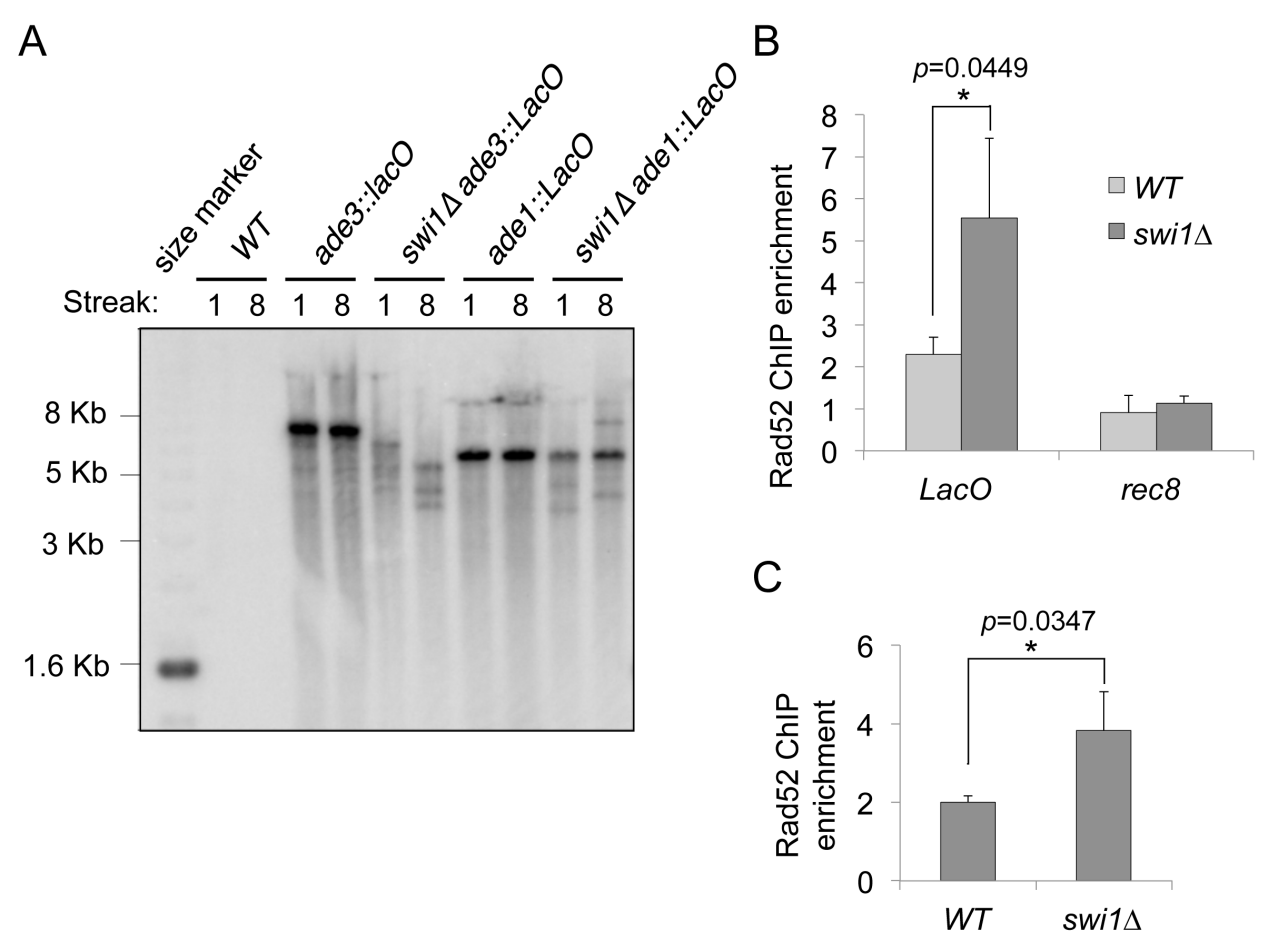
Swi1 is necessary for accurate replication of *LacO*-repeat DNA tracts. **(A)** Southern blot analysis using a *LacO* probe on genomic DNA extracted from wild-type or *swi1Δ* cells carrying the *LacO* array. Genomic DNA was prepared after 1 or 8 restreaks after the indicated strains were generated, digested with *StuI* and separated on a 0.4% agarose gel. Similar results were obtained with another independent *swi1Δ ade1*::*LacO* strain. A wild-type strain with no *LacO* repeats was used as a control for potential background hybridization. **(B)** Rad52 is enriched at *LacO* repeats but not at the meiotic gene *rec8* where no replication obstacles are expected. ChIP assays of Rad52-12Pk were performed on cell extracts prepared form the indicated cells. The immunoprecipitated DNA was subjected to competitive multiplex PCR to monitor the association of Rad52-12Pk with the *LacO* tract and the *rec8* gene, using the gene-free region 2 (GFR2) and GFR3 as internal amplification control loci, respectively. Chromatin association is presented as described in Fig 1C. Swi1 deletion significantly enhanced Rad52 recruitment at *LacO* repeats, while no significant difference was found at the *rec8* locus. Statistical analysis of three independent experiments was performed as described in Fig 1C. **(C)** Swi1 deletion causes DNA damage at rDNA repeats. ChIP assays show enrichment of Rad52-12Pk at rDNA repeats in wild-type and *swi1*Δ cells. *swi1Δ* cells display a 2-fold increase in Rad52 binding at rDNA compared to wild-type. ChIP and competitive multiplex PCR were performed using primers specific for the rDNA (rDNA_2) and a gene-free region (GFR2). Statistical analysis of three independent experiments was performed as described in Fig 1C.

Immediately after the *swi1* deletion was introduced into the strains that carry the *LacO* array, segregants were passaged by restreaking multiple times on YES plates in order to stabilize repeat length. Southern blot analyses using a *LacO* repeat-specific probe show that wild-type cells maintained the *LacO* repeat length (~8 kb and ~6 kb in strains carrying the *LacO* array at *ade3* and *ade1* loci, respectively) even after cells were passaged multiple times (Fig 3A). In contrast, *swi1Δ* cells displayed dramatic shortening of *LacO* repeats integrated at the *ade3* locus indicative of repeat instability. The effect of *swi1* depletion on *LacO* repeat instability was clear at the earliest passage and became more prominent with the consecutive streaks. We also observed considerable repeat instability that resulted in both longer and shorter *LacO* repeats when the construct was integrated at the *ade1* locus, suggesting that Swi1 is involved in replication and/or maintenance of repeat DNA sequences independently of the repeat location (Fig 3A). Such instability appears to cause DNA damage as *swi1* deletion resulted in a significant enrichment of Rad52 at the *LacO* repeats while no difference was found at a non-repetitive locus such as *rec8* (Fig 3B). We also found similar results at rDNA repeat regions, where loss of *swi1* caused increased DNA damage as represented by elevated recruitment of Rad52 at rDNA repeats (Fig 3C). This is consistent with the fact that *swi1*Δ cells have a shorter chromosome III [67–70], which contains rDNA repeats in *S*. *pombe*. These results suggest that Swi1 is required for preventing DNA damage at repeat DNA regions. In particular, our findings indicate that Swi1 plays a critical role in proper replication of *LacO* as well as rDNA repeats.

**Fig 4:**
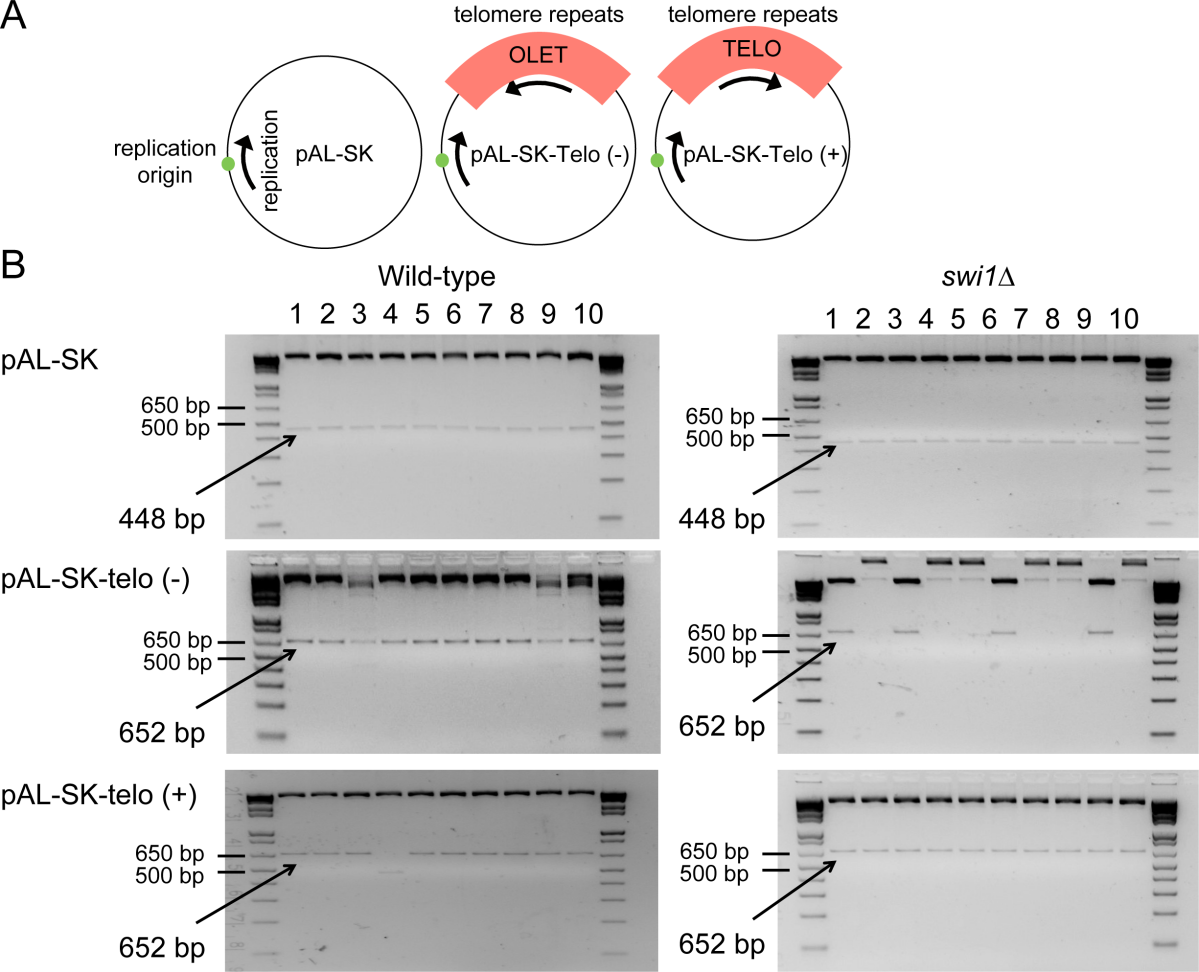
Plasmid instability associated with telomeric repeats. (A) Schematic diagram of pAS-SK-telo plasmids. (B) Wild-type and *swi1Δ* cells were transformed with either the empty pAL-SK plasmid or the pAL-SK carrying a 300 bp. telomere tract in normal (+) or reverse (-) orientation relative to the replication origin of the plasmid. Recovered plasmid from 10 yeast colonies was amplified in bacteria, and 10 bacterial colonies per treatment were analyzed by restriction digestion with *PvuII* in 3% agarose gels stained with EtBr. Each column represents and independent bacterial colony. Size markers are shown and expected restriction fragment size is shown in bold.

The aforementioned results suggest that repetitive DNA presents a replicative obstacle in *swi1*Δ cells. Since telomeres are composed of highly repetitive DNA, we hypothesized that telomere shortening observed in *swi1*Δ cells could be attributable to the repetitive nature of telomere DNA. The presence of DNA ends at telomeres could complicate interpretation of our analysis because the end-replication problem, telomerase recruitment, and recombination among chromosome ends can all affect telomere length. Thus, to specifically investigate contribution of Swi1 in maintaining telomere repeat stability, we studied the stability of a single synthetic internal telomere tract (~300 bp) within an episomal plasmid (Fig 4A).

We generated pAL-SK plasmids carrying a single 300-bp telomere tract (*leu1*::*telomere)* inserted in either forward (+) or reverse (-) orientation with respect to the position of the replication origin in the plasmid. These plasmids represent the natural (+) and inverse (-) orientation of the telomeric repeats in reference to the replisome movement (Fig 4A). To allow for plasmid replication in the presence or absence of Swi1, wild-type and *swi1*Δ cells were transformed with either the empty plasmid or the plasmid carrying the telomere tract in either orientation. Recovered plasmid was amplified in bacteria, and 10 bacterial colonies per treatment were analyzed by restriction digestion with *Pvu*II.

As shown in Fig 4B, both wild-type and *swi1*Δ mutant strains were able to amplify the empty plasmid as all colonies analyzed displayed the expected restriction fragment length. Interestingly, the restriction pattern of the pAL-SK-telo(-) plasmid obtained form *swi1*Δ cells was significantly altered in six out of the 10 colonies analyzed, while the same plasmid obtained from wild-type cells showed the expected band size in all cases. Restriction analysis of the recovered pAL-SK-telo(+) plasmids showed the expected fragment lengths in most of the colonies from both wild-type and *swi1*Δ mutants strains (Fig 4B). Although these results might seem contradictory as the pALSK-telo(+) plasmid carries the native repeat orientation, it is important to note that we obtained a very low number of colonies for *swi1*Δ cells transformed with the pALSK-telo(+) plasmid (<10 *E*. *coli* colonies per transformation) in comparison to all other treatments (50–100 colonies per transformation). One possible explanation of this observation is that replication of the pAL-SK-telo(+) plasmid could generate toxic intermediates that are unfavorable for growth of *swi1*Δ cells. It is likely that, as a result of this toxicity, the colonies obtained represent a small subset of yeast cells that carried an unaltered pAL-SK-telo(+) plasmid. Nevertheless, these results are consistent with a notion that Swi1 is critical for replication of repetitive DNA sequences, independently of location and DNA sequence. Furthermore, our data suggests that telomeric repeats, in either orientation, are a significant replication obstacle for Swi1-depleted cells, but their presence in native conformation might be most detrimental for cells. Our data are in agreement with experiments done with trinucleotide repeats where it was shown that the stability of trinucleotide repeats in bacterial, yeast, and cultured mammalian cells depend on their orientation with regard to replication origins [71–74]. Thus, our results indicate that repetitive DNA at telomeres is an important source of instability in the absence of Swi1.

### Heterochromatin-related proteins and shelterin are not major obstacles for DNA replication in the absence of Swi1

Similar to the endogenous telomere ends, the telomere tract in the pAL-SK-telo plasmids can recruit telomere-binding proteins such as shelterin components and heterochromatin [75]. Therefore, it was possible that replisome progression through telomeric repeats might be blocked by telomere-binding factors such as shelterin components and heterochromatin related proteins in *swi1Δ* cells. If so, removal of these features from telomeres would rescue the telomere shortening phenotype of *swi1*Δ cells.

**Fig 5:**
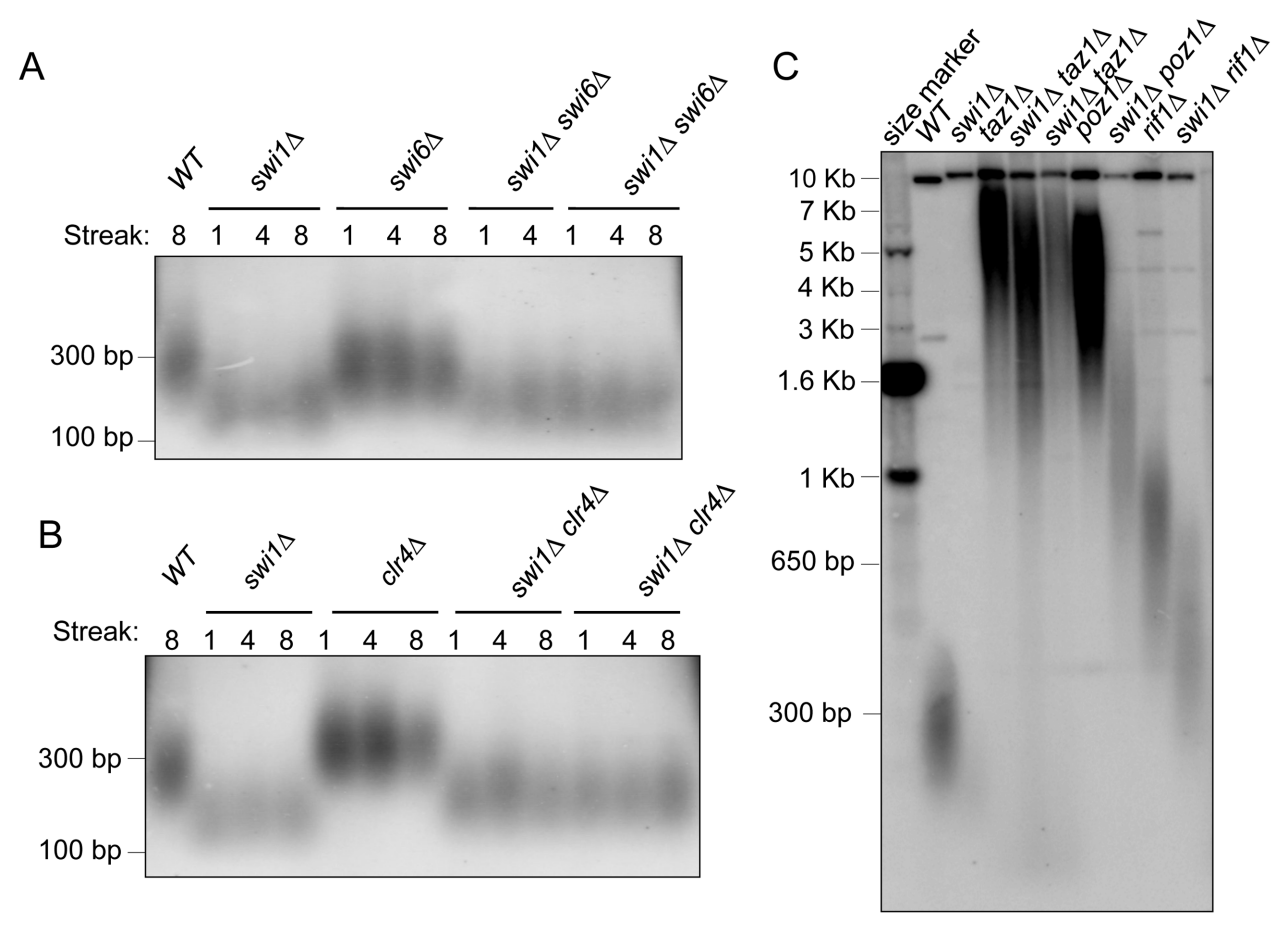
Neither heterochromatin disruption nor shelterin removal can rescue telomere shortening in *swi1Δ* cells. **(A-B)** Loss of heterochromatin-related proteins does not rescue telomere-shortening phenotype of *swi1*Δ cells. Southern blot analysis of telomere fragments from the indicated mutants. At least two independent segregants of the double deletion mutants were examined. Genomic DNA was prepared after 1, 4, or 8 restreaks after the indicated strains were generated. *Apa*I-telomere fragments were detected using a telomere-specific probe as described in Fig 2B. **(C)** Telomere shortening still occurs in the absence of shelterin components when *swi1* is deleted. Cells with the indicated genotypes were passaged at least 8 times and subjected to Southern blot analysis as described in Fig 2B.

To disrupt heterochromatin, we deleted genes required for heterochromatin formation. These genes include *swi6*^+^ and *clr4*^+^, which encode proteins homologues to human HP1 [76, 77] and Suv39 family of histone methyltransferases [78–80], respectively. *swi6* or *clr4* deletion failed to shorten telomere length in southern blots of *ApaI* fragments (Fig 5A and 5B), in agreement with related studies [81, 82]. When Swi1 was removed from *swi6Δ* or *clr4*Δ cells, telomeres were shortened to the extent of the telomere length in *swi1*Δ cells (Fig 5A and 5B). Thus, disruption of these HP1-related heterochromatin proteins failed to alleviate telomere shortening of *swi1*Δ cells. These results suggest that heterochromatin does not cause observable telomere damage and thus does not present a severe obstacle to the DNA replication process.

The shelterin complex, which directly associates with telomeres, has been shown to facilitate replication of telomeric repeats in fission yeast cells [39, 60]. However, previous studies in budding yeast have reported that telomere-binding proteins can become a barrier to DNA replication process [31, 83]. Because the replisome is unstable in *swi1Δ* cells, we tested whether shelterin could act as a replication obstacle in the absence of Swi1. Loss of shelterin components including Taz1, Poz1, and Rif1 resulted in telomere lengthening when compared to wild-type cells as seen in *Apa*I-telomere length analysis (Fig 5C). This is consistent with previous findings showing that shelterin can act as a negative regulator of telomerase [84–86], as well as with a protective role of Taz1 against activation of homologous recombination (HR)-based telomere lengthening [87, 88]. We then determined telomere length of *swi1*Δ *taz1*Δ, *swi1*Δ *poz1*Δ, and *swi1*Δ *rif1*Δ strains. *swi1* deletion led to considerable telomere shortening in *poz1Δ* and *rif1Δ* cells and less dramatic but still significant telomere shortening in *taz1*Δ (Fig 5C), indicating that Swi1 is still important for telomere length maintenance even in the absence of shelterin subunits that inhibit telomere extension.

### Swi1 plays a telomerase-independent role in promoting telomere repeat length maintenance

**Fig 6:**
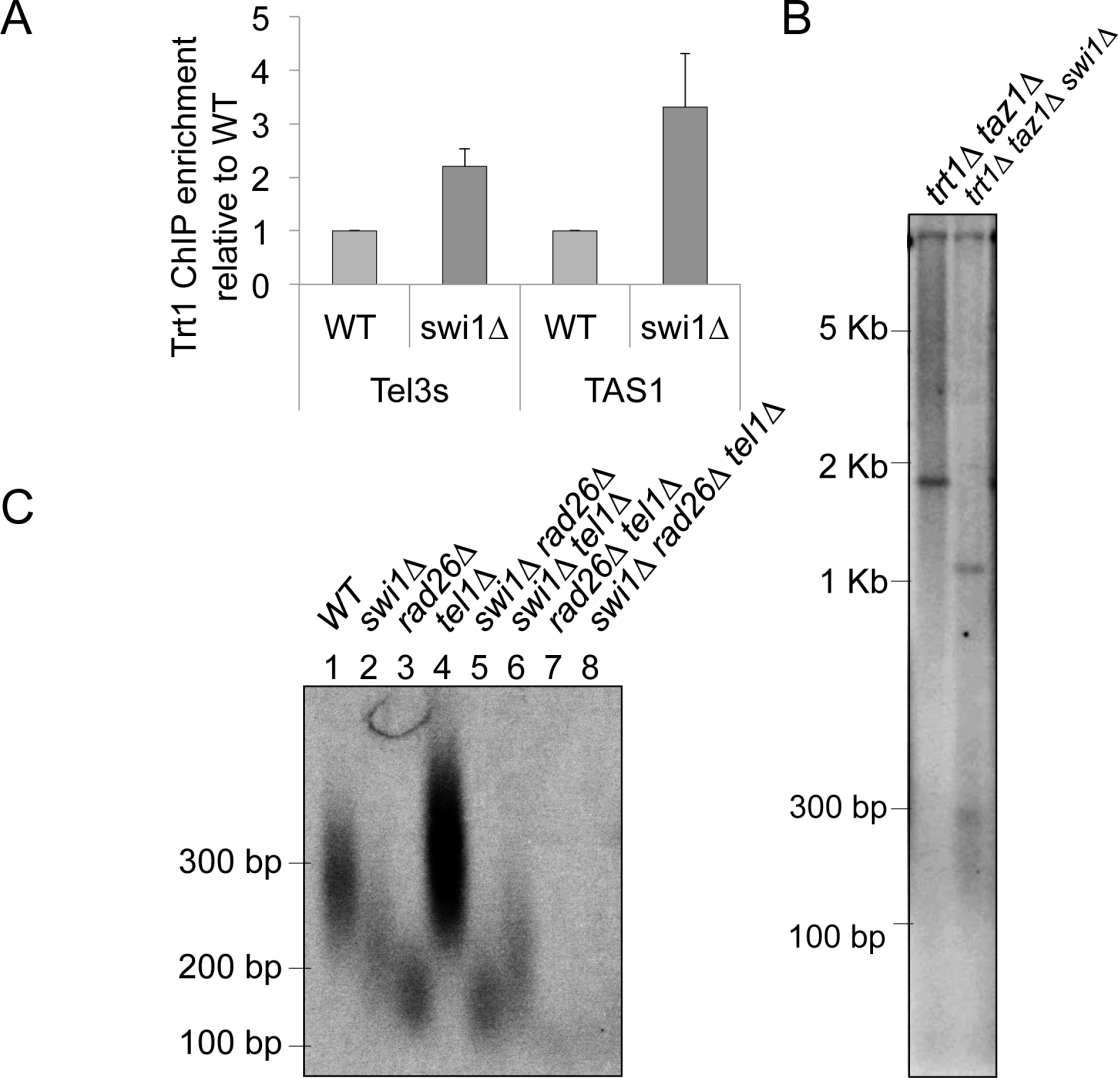
Telomere shortening in *swi1Δ* mutants is not caused by defects in telomerase recruitment at telomeres. **(A)** Strains of the indicated genotypes were engineered to express Trt1-G9-5FLAG [50]. ChIP results show that telomerase is preferentially recruited at short telomeres in *swi1Δ* mutants compared to wild-type length telomeres. Telomerase recruitment was monitored by competitive multiplex PCR at two different subtelomeric regions: Tel3s and TAS1. GFR1 (for Tel3s) and GFR2 (for TAS1) were amplified as internal control regions in multiplex PCR reactions. Trt1 enrichment at Tel3s and TAS1 over control regions was determined. Data is shown as relative fold enrichment in comparison to WT. Error bars represent the standard deviation obtained from three independent experiments. **(B)** *trt1Δ taz1Δ* and *trt1Δ taz1Δ swi1Δ* cells were passaged at least 8 times, and Southern blot analysis of *Apa*I-digested genomic DNA was performed as described in Fig 2B. **(C)** Genetic interaction between Swi1 and Rad3-Rad26 in telomere maintenance. Genomic DNA was isolated form the indicated cells, and telomere southern analysis was performed as described in Fig 2B.

Our analysis of telomere-repeat stability within episomal plasmids in *swi1Δ* cells (Fig 4) suggested that inability of *swi1*Δ cells to maintain stable repetitive telomere repeats may contribute to telomere shortening in *swi1*Δ cells. However, since telomerase recruitment is coupled to replication of telomeres by replicative DNA polymerases, it was possible that loss of FPC might prevent efficient recruitment of telomerase, leading to telomere shortening. Thus, we decided to examine the recruitment of telomerase to telomeres in *swi1*Δ cells. As shown in Fig 6A, ChIP assays revealed that Trt1 recruitment was significantly elevated in *swi1*Δ cells when compared to wild-type cells. This is consistent with previous studies showing that telomerase is selectively recruited at shorter telomeres in fission yeast [60].

Interpretation of epistasis analysis between *swi1Δ* and shelterin subunit mutant (*taz1Δ*, *poz1Δ* and *rif1Δ*) is complicated due to the fact that telomerase can indeed still act on telomeres to extend telomeres in the absence of Swi1. Thus, to evaluate whether the Swi1 indeed plays a telomerase-independent role in maintaining telomere length, we then sought to evaluate effect of eliminating Swi1 in cells that lack telomerase. Since telomerase (*trt1*^+^) deletion frequently leads to telomere loss and generation of survivor cells that eliminate telomeres by circularizing chromosomes [87], we utilized a *taz1*Δ *trt1*Δ double mutant strain, which stably maintains heterogeneous telomeres via recombination-dependent, ALT-like telomere maintenance mechanism [87, 88].

Telomeres in *taz1*Δ *trt1*Δ cells were much longer than those in wild-type cells, which is consistent with the ALT-like phenotype of *taz1*Δ *trt1*Δ cells (Fig 6B). Importantly, *swi1*Δ *taz1*Δ *trt1*Δ triple mutant cells carried shorter telomeres than *taz1*Δ *trt1*Δ cells, suggesting that telomere shortening due to *swi1* deletion is not caused by a defect in telomerase activity. Interestingly, *swi1*Δ cells rapidly undergo telomere shortening within the first passage after *swi1* deletion, and the telomere length becomes stable afterwards. In striking contrast, *trt1Δ* cells displayed a much slower and progressive telomere shortening (S2 Fig) [89]. Taken together, we conclude that Swi1 is involved in telomere length maintenance independently of telomerase activity.

Rad3-Rad26 and Tel1-MRN (Mre11-Rad50-Nbs1) kinase complexes are redundantly required for telomerase recruitment at telomeres by promoting phosphorylation of the threonine 93 residue of Ccq1 [90, 91]. Thus, in the absence of Tel1, Rad3-Rad26 becomes essential for telomerase recruitment as *rad3*Δ *tel1*Δ and *rad26*Δ *tel1*Δ double mutants lose telomeres [56, 58]. To understand the relationship between Swi1 and Rad3-Rad26, we constructed *swi1*Δ *tel1*Δ double mutants cells. If Swi1 contributes to Rad3-Rad26-dependent telomerase recruitment, we expect that *swi1*Δ *tel1*Δ cells would have shorter telomeres than *swi1*Δ cells. However, *tel1* deletion had no effect on the telomere length of *swi1*Δ cells (Fig 6C), suggesting that Swi1 is unlikely to be required for Rad3-Rad26’s ability to promote telomerase recruitment. Such conclusion is entirely consistent with our earlier ChIP analysis, which suggested that Swi1 is not necessary for telomerase recruitment (Fig 6A).

On the other hand, we found that telomere length of *swi1*Δ *rad26*Δ cells is similar to that of either single mutant (Fig 6C), suggesting that the telomere length maintenance defect observed in *rad26Δ* is epistatic to *swi1Δ*. Since previous genetic analysis indicated that Rad3-Rad26 contributes to telomere protection function that appears to be distinct from Rad3-Rad26’s role in telomerase recruitment [92], we suggest that Rad3-Rad26’s ability to protect telomeres is mediated by Swi1.

### Short telomeres in *swi1*Δ cells are maintained by telomerase and not by homologous recombination

**Fig 7:**
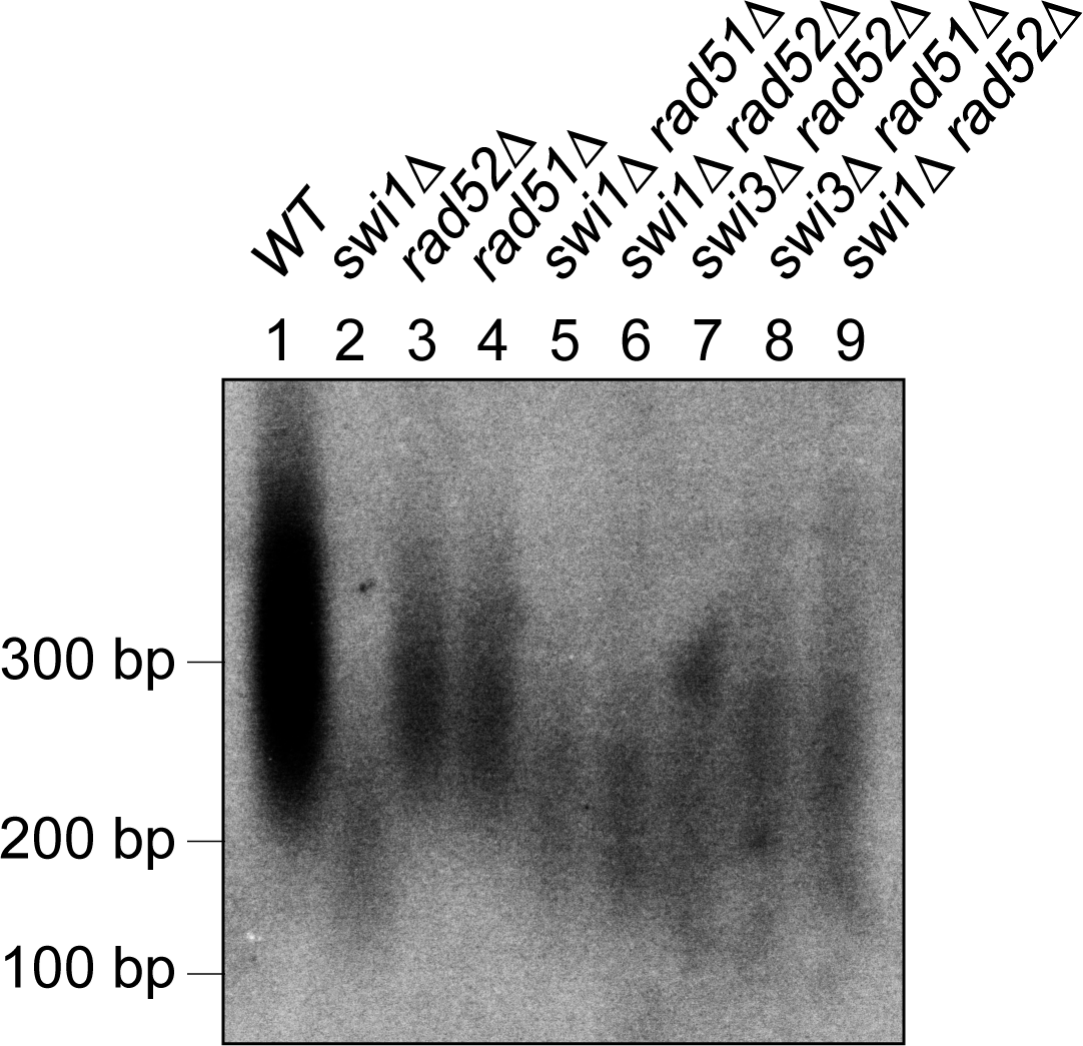
Homologous recombination is not involved in maintaining telomeres in *swi1*Δ cells. HR proteins are dispensable for maintaining telomeres in *swi1Δ* cells. Telomere southern analysis of the indicated strains was performed as described in Fig 2B.

Although telomeres rapidly shorten after the loss of Swi1, *swi1*Δ cells do not lose all telomeric repeats. Instead, they stably maintain short telomeres even after extensive passages. Because Rad52 recruitment is increased in *swi1*Δ cells, it was possible that recombinational telomere maintenance pathways might maintain telomeres in the absence of Swi1. Therefore, we inactivated homologous recombination (HR) by deleting *rad51*^+^ or *rad52*^+^ and determined the effect of these deletions on telomere length. As shown in Fig 7, telomere length of *rad51*Δ and *rad52*Δ cells was similar to that of wild-type cells. Importantly, when we deleted *rad51*^+^ or *rad52*^+^ from *swi1*Δ or *swi3*Δ cells, cells still showed short telomeres similar to those in *swi1*Δ cells (Fig 7). These results indicate that HR is not the major pathway used to maintain short telomeres in *swi1Δ* cells and further suggest that telomerase is functional in *swi1Δ* cells.

### Deletion of *swi1* causes an increase in emergence of ALT-like survivors with amplified telomere/subtelomere regions in the absence of telomerase

**Fig 8:**
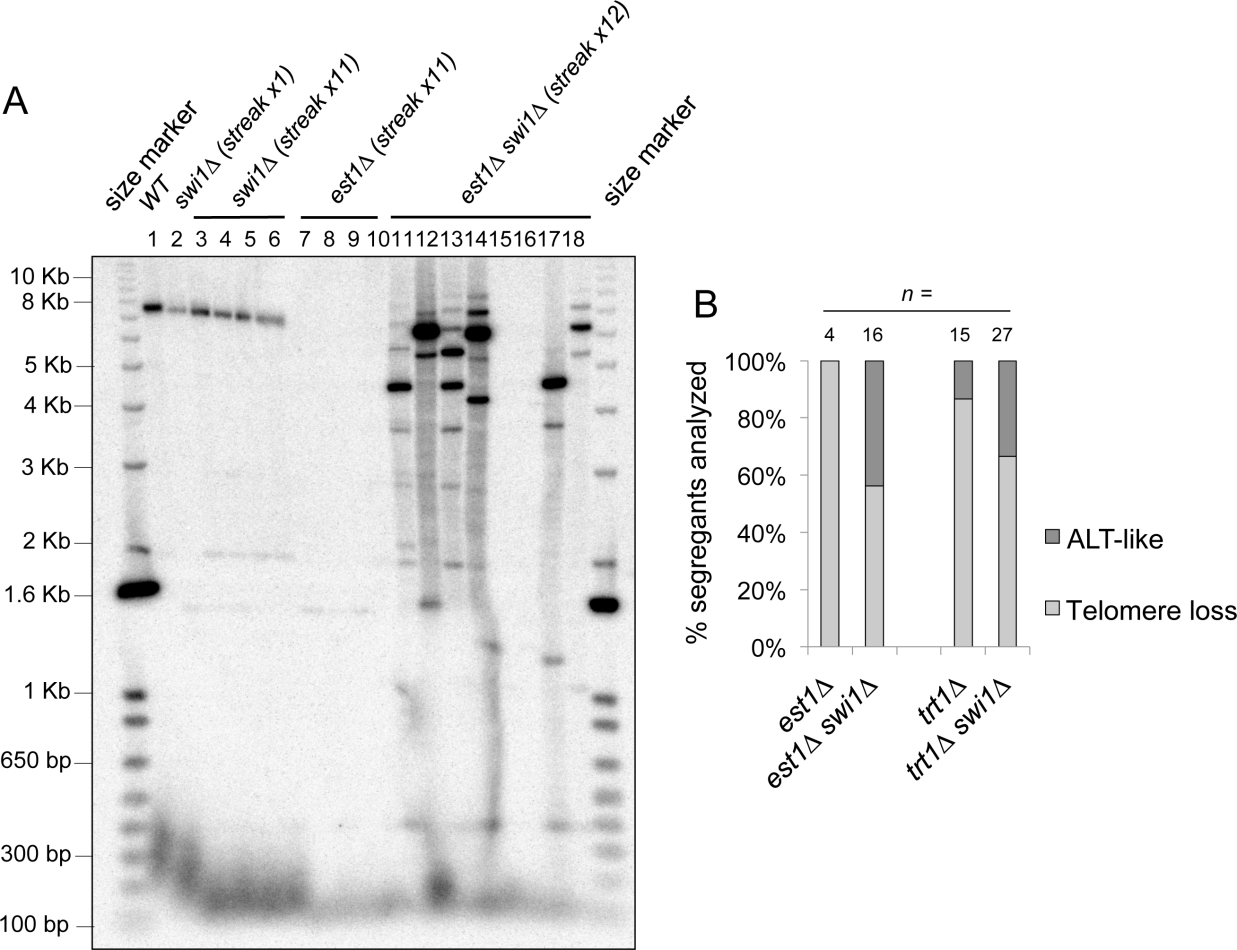
Swi1 is required to prevent hyperrecombination at telomeres and ALT-like telomere phenotypes in the absence of telomerase. **(A)** *swi1* deletion significantly increases the chances of telomerase negative cells to undergo ALT-like telomere maintenance. Representative image of telomere Southern blot analysis of wild-type, *swi1*Δ, *est1*Δ and *est1*Δ *swi1*Δ cells. Multiple segregants with the same genotypes were analyzed after at least 11 passages. *Apa*I-digested DNA was hybridized with the telomere-specific probe as described in Fig 2B. Representative results are shown. **(B)** Quantification of the independent segregants analyzed for each genotype and classification depending on their telomere phenotype.

The elevated recruitment of telomerase in *swi1*Δ cells and the ability of these cells to maintain telomeres in HR-deficient backgrounds suggest that short but stable telomeres in *swi1*Δ cells are maintained by telomerase. This notion prompted us to investigate the physiological consequences of telomerase loss in *swi1*Δ cells. To inactivate telomerase, we first utilized an *est1*-deletion background because the telomerase regulatory subunit Est1 is essential for telomerase-dependent telomere maintenance in fission yeast [93]. We deleted *swi1*^*+*^ from *est1*^+^/*est1*Δ diploid cells and generated a *swi1*^+^/*swi1*Δ *est1*^+^/*est1*Δ diploid strain, from which we obtained haploid *est1*Δ, *swi1*Δ and *est1*Δ *swi1*Δ strains. As expected from previous studies [93], all *est1*Δ strains underwent extreme telomere shortening (Fig 8A and 8B). When telomere length of multiple *swi1*Δ *est1*Δ strains was monitored, some strains also had extremely short telomeres. However, a strikingly higher proportion of *swi1*Δ *est1*Δ strains developed long and heterogeneous telomeres (Fig 8A and 8B), a feature reminiscent of ALT activation in humans [94, 95] and type-II survivors in *S*. *cerevisiae* and *Kluyveromyces lactis* [96–98].

To further confirm this phenomenon, we also deleted telomerase catalytic subunit *trt1*^*+*^. Because we were unable to obtain viable *swi1Δ trt1Δ* cells when we attempted to obtain double mutant cells by gene deletion or genetic cross of *trt1Δ* and *swi1Δ* strains, we chose to cross *swi1*Δ cells with *trt1*Δ *taz1*Δ cells to obtain viable *swi1*Δ *trt1*Δ cells. Wild-type, *swi1*Δ, *taz1Δ* and *swi1Δ taz1Δ trt1Δ* segregants obtained from the tetrad dissection displayed the expected telomere phenotypes (S3 Fig). Interestingly, again, many of *swi1*Δ *trt1*Δ double mutant strains showed long and heterogeneous telomeres whereas most of *trt1Δ* mutants had extremely short telomeres or lost telomeres (Fig 8B and S3 Fig). The number of ALT-like telomeres observed in the *trt1Δ* strains was higher than expected from *trt1*-deficient strains (Fig 8B). This is probably due to the presence of long telomeres in the *taz1Δ trt1Δ* strain that was used to generate *trt1*Δ segregants. Taken together, these results suggest that, in the absence of telomerase, short telomeres caused by replication fork instability become templates for recombination-dependent (ALT) maintenance of telomeres. Our findings also suggest that Swi1 protects telomeres from hyper-recombination when telomerase is absent.

## Discussion

In this work, we identified a novel role of Swi1 in preventing repeat instability during DNA replication in *S*. *pombe*. More specifically, our investigation revealed that loss of FPC causes telomere instability during replication, which may contribute to telomere shortening in *swi1Δ* cells. Telomeric repeats rather than other telomeric features present a significant obstacle for the replisome and thus render telomeres difficult to replicate. In addition, our results also suggest that Swi1^Timeless^ prevents recombination-dependent ALT-like telomere amplification in the absence of telomerase. Based on these results, we propose that the role of the FPC could be further exploited in the context of mammalian models as a potential link between genomic instability and ALT-dependent tumorigenesis.

### The FPC’s role in telomere replication and telomere length maintenance

Swi1 deletion renders *S*. *pombe* cells highly sensitive to fork-stalling agents and causes extensive accumulation of spontaneous Rad52 DNA repair foci during S phase, indicating that Swi1 stabilizes stalled replication forks [47]. Rad52 foci may represent DNA damage at specific chromosome loci that are more difficult to replicate when Swi1 is depleted. These loci include rDNA repeats and telomeres as they are enriched with Rad52 (Fig 1C, Fig 3C). In addition, *swi1*Δ cells accumulate broken forks at rDNA loci and show elevated levels of TIFs (Fig 1D and 1F) [47]. We therefore suggest that these genomic regions are among the hot spots of DNA damage in *swi1*Δ cells.

It is possible that stalled or slowed forks at rDNA- or telomere-repeat regions need to be stabilized by Swi1, in order to prevent fork collapse. Importantly, Swi1 coordinates leading- and lagging-strand synthesis at the replication fork [47, 70]. Furthermore, the recruitment of the lagging-strand polymerase (Pol δ) at telomeres is significantly delayed when compared to the leading-strand polymerase (Pol ε) in wild-type cells [52, 59, 60]. Therefore, we propose that loss of Swi1 leads to extensive uncoupling of leading- and lagging-strand synthesis, causing telomeres to adopt unusual open configurations that are prone to fork collapse and DNA damage.

**Fig 9:**
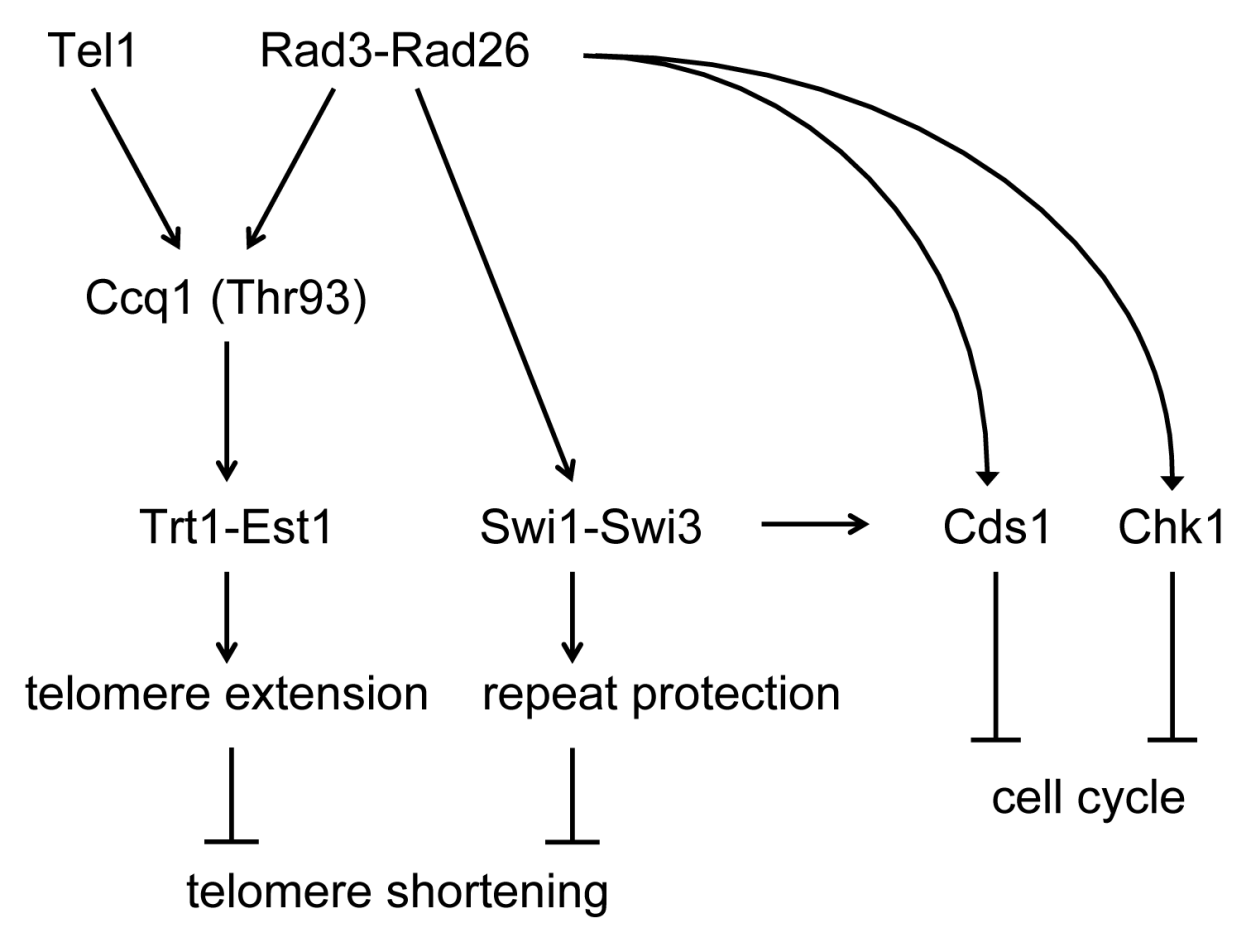
Model of the Swi1-Swi3 FPC-dependent telomere length maintenance. For details, see text.

Telomere damage was linked to the occurrence of telomere shortening in *swi1*Δ cells. Telomere length in *swi1Δ* strains was comparable with that in *rad3*Δ and *rad26*Δ cells. In fission yeast, Rad3^ATR^ and Rad26^ATRIP^ form a complex that is essential for cell cycle arrest by activation of both Cds1^Chk2^ and Chk1^Chk1^ kinases responsible for the DNA replication and damage checkpoints, respectively [99]. Swi1 is also involved in full activation of the Cds1 checkpoint kinase in *S*. *pombe* [47]. However, our epistasis analysis indicates that both Swi1-Swi3 and Rad3-Rad26 complexes maintain telomere length independently from their role in the activation of downstream checkpoint kinases Cds1 and Chk1 (Fig 9). Our genetic data also suggest that the FPC and Rad3-Rad26 are in the same pathway for telomere length maintenance (Fig 9). Although Rad3-Rad26 phosphorylates the threonine 93 residue of Ccq1 to facilitate telomerase recruitment at telomeres [90, 91], Swi1 appears to play no role in telomerase recruitment as *swi1*Δ cells showed an increased level of telomerase at telomeres (Fig 6A). Since our previous genetic analysis indicated that Rad3-Rad26 contributes to telomere protection [92], and this protective function appears to be distinct from it’s role in telomerase recruitment, we suggest that telomere protection function of Rad3-Rad26 is medicated by Swi1 (Fig 9).

### Identifying the telomeric replicative obstacles in FPC deficient cells

*swi1* deletion causes contraction of rDNA repeats [68, 70]. In this study, we demonstrated that *swi1*Δ cells harbor short telomeres and undergoes repeat loss and instability associated with the *LacO* array, indicating that the primary replicative obstruct in *swi1*Δ cells is repeat DNA sequence.

In *E*. *coli*, *LacO* arrays bound by the LacI protein block replication by forming a site-specific protein–DNA complex that serves as an RFB [100, 101]. In mammalian cells, insertion of *LacO* repeats generates fragile sites in metaphase chromosomes [102]. This also appears to be dependent on LacI repressor proteins that form RFBs by binding to the *LacO* repeats [103]. Furthermore, studies in *S*. *pombe* showed that the LacI-*LacO* system leads to replication fork block and DSBs [66], further suggesting the role of protein-DNA complexes in replication interference. Consistently, in our present study, *LacO* arrays induced only a mild increase in Rad52 recruitment when LacI was not present in wild-type cells. However, Rad52 was further accumulated at *LacO* repeats in *swi1*Δ cells even in the absence of LacI (Fig 3B), suggesting that Swi1 is required for replication of repeat DNA. Considering that the induction of DNA damage response at the *LacO* arrays bound by LacI occurs as a consequence of DNA replication [103], the repetitive nature of repeat sequences poses an initial layer of replication problems that is counteracted by Swi1. As in the case of the LacI-*LacO* RFB, protein-DNA RFBs may provide another layer of replication stress that further complicates replisome passage even in wild-type cells.

Although our studies demonstrated that repetitive DNA is the primary replication problem in the absence of Swi1, it is important to note that several other characteristics of the telomeres may further complicate telomere replication. In budding yeast, the replication fork pauses at telomeres, and this pausing is intensified in the absence of Rrm3 helicase [31]. Furthermore, the presence of Rap1 bound to the telomeric sequences has been suggested to be a component of the telomere replication barrier [30]. In fission yeast, Taz1 is required for efficient telomere replication, and Poz1 and Rap1 also contribute to the completion of telomere replication by promoting timely accumulation of the lagging-strand DNA polymerase and the Stn1-Ten1 complex at telomeres [30, 31, 39]. In addition, the presence of heterochromatin and the potential to form t-loop structures may provide an additional barrier to the passage of the fork [104]. Therefore, further research is necessary to fully understand the composition of the telomeric barrier in fission yeast as well as the replication factors involved in overcoming this barrier. Future studies should focus on the mechanisms by which these telomere features add additional layers of difficulty to the passage of the replication fork.

### Possible implications for a role of the mammalian FPC in telomere regulation

Approximately 10–15% of cancer cells including glioblastomas and osteosarcomas [44] are able to escape replicative senescence by activating the recombination-dependent ALT pathway in the absence of any detectable telomerase activity [105]. Although the prevalence of ALT telomeres is well established, the factors or events that trigger certain cancer cells to activate ALT pathways over telomerase reactivation are not well understood. Accordingly, there are no treatments targeting ALT specifically in cancer cells and, in addition, these tumors are predicted to be resistant to anti-telomerase therapies [106].

Our results show that Swi1-deficient cells display increased association of Rad52 recombinase, and that loss of Swi1 promotes recombination-based survival in cells lacking telomerase. Therefore, telomere damage during DNA replication may enhance DNA repair processes using the homologous recombination machinery. It is possible that this pathway provides a more robust and efficient response to drastic telomere shortening than telomerase reactivation, in order to maintain telomere length after a rapid and significant telomere repeat loss.

A recent paper showed that cancer cells bearing ALT telomeres are sensitive to ATR inhibitors. This is because ALT telomeres have elevated levels of RPA-coated ssDNA, which is known to recruit ATR [106]. Our findings in *S*. *pombe* are in agreement with this work, as loss of Swi1 generates long stretches of ssDNA at the stalled forks [21, 70]. Consistently, Chk1, an effector kinase downstream of Rad3^ATR^ is activated in *swi1*Δ cells [47]. It is therefore conceivable that Rad3^ATR^ activation in Swi1-deficient cells mediates the formation ALT telomeres observed in *swi1Δ est1Δ* and *swi1Δ trt1Δ* cells. Future investigations are warranted to test this possibility and further address the role of Swi1^Timeless^ in preventing ALT-dependent cancers.

## Materials and Methods

### General techniques

The methods used for genetic and biochemical analyses of fission yeast have been previously described [107, 108]. For telomere length assays, yeast cells were grown at 32°C on solid YES (yeast extract with supplements) plates. To ensure stabilization of the telomere length, cells were streaked on YES plates every 3 days at least 8 consecutive times prior to genomic DNA preparation. Microscopic analyses of fluorescent proteins, Western blotting, and drug sensitivity assays were performed as described in our earlier studies [21, 47, 68, 109].

### *S*. *pombe* strains and plasmids

The *S*. *pombe* strains used in this study were constructed using standard techniques [107, 108], and their genotypes and sources are listed in S1 Table. To construct episomal plasmids that carry a telomere tract, a 300 bp *S*. *pombe* telomere fragment was subcloned from pTELO plasmid [60] into the *Pvu*II site of the pAL-SK plasmid [110], which carries the *LEU2* gene, resulting in pAL-SK-Telo (+) and pAL-SK-Telo (-). To express Rad52-YFP (originally called Rad22, but recently renamed as Rad52 in *S*. *pombe*) as a sole source of Rad52 from the native promoter, pJK210-Rad52CT-YFP [68] was linearized at the *Afl*II site, which is localized within the DNA sequence that encodes the C-terminal (CT) domain of Rad52, and inserted at the *rad52* locus of *S*. *pombe* strains. *rad52*Δ (*rad52*::*hphMX6*) was generated by a two-step PCR method [111], to replace the *rad52* open reading frame with the *hphMX6* gene. *cds1*Δ (*cds1*::*hphMX6*) was generated by a one-step marker switch method [112] using *cds1*::*kanMX6* [67]. *taz1-mCherry* (*taz1-mCherry*::*hphMX6*) was also generated by the two-step PCR method, to construct a mCherry tag at the C-terminus of *taz1*. Plasmids used as template for the PCR-based gene disruption and gene tagging have been previously described for pFA6a-hphMX6 [113] and pFA6a-5FLAG-kanMX6 [114]. pFA6a-mCherry-hphMX6 was constructed by replacing the *Bgl*II-*Eco*RI *kanMX6* fragment in pFA6a-mCherry-kanMX6 [115] with *hphMX6* fragment. pJK148-Swi1 was constructed by inserting the 3.6 kb genomic fragment containing the *swi1*^+^ gene into the *Sac*I/*Bam*HI site of pJK148 [116].

Mutations and epitope-tagged genes have been previously described for *swi1*Δ (*swi1*::*kanMX6*, *swi1*::*natMX6*) [47, 69], *rad3*Δ (*rad3*::*ura4*^+^, *rad3*::*kanMX6*) [67, 117], *rad26*Δ (*rad26*::*ura4*^+^) [118], *tel1*Δ (*tel1*::*ura4*^+^, *tel1*::*LEU2*) [56], *cds1*Δ (*cds1*::*ura4*^+^) [119], *chk1*Δ (*chk1*::*ura4*^+^, *chk1*::*kanMX6*) [67, 118], *clr4*Δ (*clr4*::*kanMX6*) [79], *bdf2*Δ (*bdf2*::*hphMX6*) [120], *taz1*Δ (*taz1*::*ura4*^+^, *taz1*::*LEU2*) [85], *trt1*Δ (*trt1*::*his3*^+^) [89], *rad52*Δ (*rad52*::*LEU2*) [121], *swi1-5FLAG* (*swi1-5FLAG*::*kanMX6*), *swi1-13Myc* (*swi1-13Myc*::*kanMX6*), *rad52-YFP* (*rad52-YFP*::*ura4*^+^) [21], *rad52-12PK*, *trt1-G11-9FLAG* [50], *ade3*::[*kan*^r^-*ura4*^+^-*lacO*], *ade1*::[*kan*^r^-*ura4*^+^-*lacO*] [62, 63]. Strains containing the following gene deletions were obtained from National BioResource Project Japan and used to generate various strains used in this study: *swi6*Δ (*swi6*::*kan*^*r*^, FY13724), *est1*Δ (*est1*::*kanMX*, FY14265), *poz1*Δ (*poz1*::*hyg*, FY18508), and *rif1*Δ (*rif1*::*ura4*^+^, FY14160).

### Southern blotting

Genomic DNA was digested overnight with the indicated restriction enzymes, and separated on an agarose gel using 1x Tris-Acetate-EDTA buffer. DNA from the agarose gel was transferred to a Hybond-XL membrane (GE Healthcare, Little Chalfont, UK) using buffer containing 0.5 M NaOH and 1.5 M NaCl. The membrane was then UV cross-linked using an XL-1000 UV Crosslinker (Spectronics, Westbury, NY) and incubated with a DNA probe labeled with [α-^32^P] dCTP. Hybridization was carried out overnight in Church buffer at 65°C as described [122]. Membranes were exposed for 2 days to a Phosphorimager screen, and detection of telomere or *LacO* repeats was done using a Storm 840 Phosphorimager (GE Healthcare). The DNA probe for the detection of telomere repeats by Southern blotting has been described previously [56]. For detecting *LacO* repeats, the 316-bp *Xba*I fragments that contain *LacO* repeats were excised from the pSV2-DHFR-8.32 plasmid [61] and used as a probe. The 448-bp *Pvu*II fragment from the pJK148 backbone [116] was used as a probe to detect the DNA fragment containing the internal telomere tract.

### Chromatin immunoprecipitation (ChIP) assay

ChIP assay and its quantification in all figures except for Fig 2D were carried out as described previously [123–125] with modifications. Briefly, exponentially growing cells were fixed in 3% paraformaldehyde, and chromatin was sheared into 500 to 700-bp fragments using a Misonix Sonicator 3000 (Qsonica, Newtown, CT). Chromatin-associated proteins were then immunoprecipitated using mouse monoclonal anti-V5/Pk SV5-Pk1 (AbD Serotec, Kidlington, UK) or anti-FLAG M2 (Sigma-Aldrich, St. Louis, MO) antibodies in combination with Protein G-coupled Dynabeads (Life Technologies, Carlsbad, CA). DNA extracted from the immunoprecipitates was subjected to PCR analysis, and the PCR products were separated on a 4% polyacrylamide gel. The gel was stained with SYBR Green I (Life Technologies) and analyzed with Storm 840 Phosphorimager (GE Healthcare). Relative enrichment of the target sequences was calculated by multiplex PCR including primers that amplify a gene-free region (GFR) [126] as internal control as described previously [123–125]. ChIP assay described in Fig 2D was performed using the anti-myc 9E10 monoclonal antibodies (Cell Signaling Technology, Danvers, MA) as previously described [90, 92, 127]. PCR primers used in our ChIP studies are listed in S2 Table.

### ChIP sequencing

ChIP sequencing was performed as described [124] with some modifications. ChIP samples from non-tag control (SP1173), *rad52-12PK* (Y4250) and *rad52-12PK swi1*Δ (Y4256) strains were obtained from asynchronous cultures as [128, 129]. Quality of the ChIP samples was assessed by PCR using primers that are designed to amplify *tel3s* (10 μM), *rDNA* (2.5 μM, 10 μM), and *rec8* (10 μM) loci. DNA amount was quantified using Qubit dsDNA HS assay kit (Invitrogen) and a Qubit 2.0 Fluorometer (Invitrogen). For each sample, 3.1 ng of DNA were used for library preparation. The library preparation for Illumina sequencing was done using NEBnext ChIP-seq Library Prep Master Mix Set for Illumina (New England Biolabs) following their protocol. Final DNA samples were quantified using Qubit dsDNA HS assay kit (Invitrogen) and a Qubit 2.0 Fluorometer (Invitrogen), and 120 ng of library DNA for each sample was submitted to the sequencing facility for analysis using Illumina HiSeq2000 (Illumina). Sequences were processed by the Illumina analysis pipeline version 1.6.1, and aligned to the fission yeast sequence (version ASM294v1.18). Data was visualized with Integrated Genome Browser [130]

### Telomere dysfunction-induced focus (TIF) analysis by fluorescent microscopy

Cells expressing Rad52-YFP and Taz1-mCherry from their own promoters were grown at 25°C in Edinburgh minimal medium (EMM) with necessary supplements until mid-log phase. Cells grown at 25°C have more stable fluorescence with lower background signals. Live-cell imaging analysis of Rad52-YFP and Taz1-mCherry localization was performed using an Olympus PROVIS AX70 microscope equipped with a Retiga EXi camera (QImaging, Surrey, BC, Canada). Images were acquired with iVision software (BioVision Technologies, Exton, PA) and analyzed with ImageJ software (National Institutes of Health, Bethesda, MD). At least 100 cells were counted for each experiment.

## Supporting Information

S1 Fig **(A)** To obtain the TIF ratio over total Rad52 foci, the number of total TIF events was divided by the total number of Rad52 foci in wild-type and *swi1*Δ cells. The TIF ratio in *swi1*Δ cells was more than double that of wild-type cells. **(B)** Representative microscopic images of the lateral localization of Taz1 and Rad52 are shown. Pink arrows indicate the occurrence of lateral localization events. These lateral cases were not considered as TIFs.
(TIF)

S2 Fig **(A-C)** Quantification of signal intensity of representative Southern blots of *ApaI* digested telomere fragments from wild-type (A), *swi1Δ* (B) and *trt1Δ* (C). Each image shows data of telomere lengths from DNA obtained after 1, 4, or 8 restreaks. Telomere fragments were detected using a telomere-specific probe as described in Fig 2B. Signal intensity quantification was performed with ImageJ software.
(TIF)

S3 Fig *swi1*Δ *trt1*Δ cells show ALT-like phonotype. Southern blot analysis of telomere fragments from the indicated mutants. Strains were obtained by tetrad dissection. Genomic DNA was prepared after 1, 4, or 8 restreaks after the indicated strains were generated. *Apa*I-telomere fragments were detected using a telomere-specific probe as described in Fig 2B. A representative result is shown.
(TIF)

S1 Table *S*. *pombe* strains used in this study. (XLSX)

S2 Table Oligonucleotide primers used in this study. (XLSX)
