# Supplementary figures and images for: Swi1Timeless Prevents Repeat Instability at Fission Yeast Telomeres
Source: PLoS Genet. 2016 Mar 18;12(3):e1005943. doi: 10.1371/journal.pgen.1005943 (PMC4798670; doi:10.1371/journal.pgen.1005943)

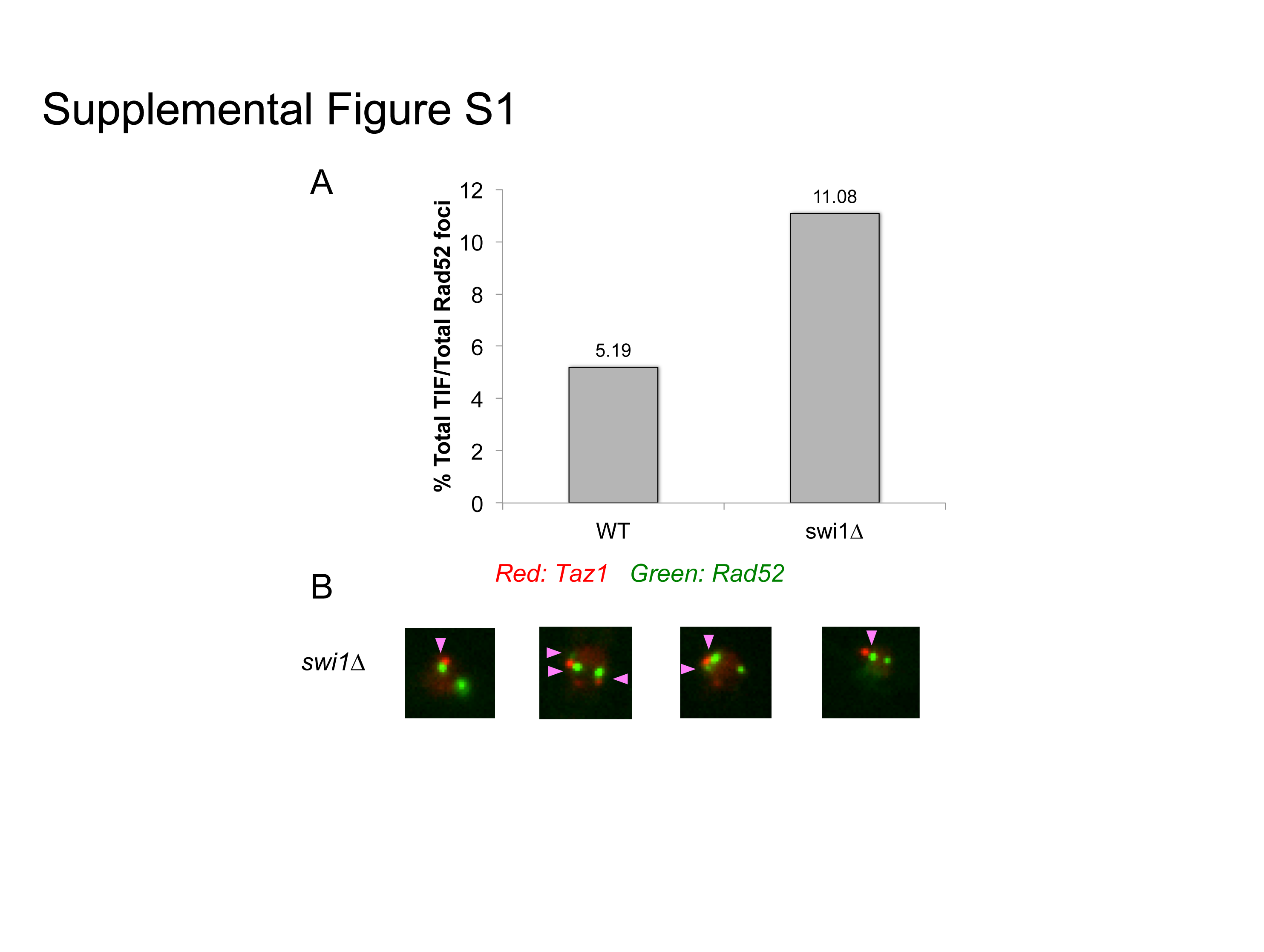

Supplement: S1 Fig — (A) To obtain the TIF ratio over total Rad52 foci, the number of total TIF events was divided by the total number of Rad52 foci in wild-type and swi1Δ cells. The TIF ratio in swi1Δ cells was more than double that of wild-type cells. (B) Representative microscopic images of the lateral localization of Taz1 and Rad52 are shown. Pink arrows indicate the occurrence of lateral localization events. These lateral cases were not considered as TIFs. (TIF) [file pgen.1005943.s001.tif]

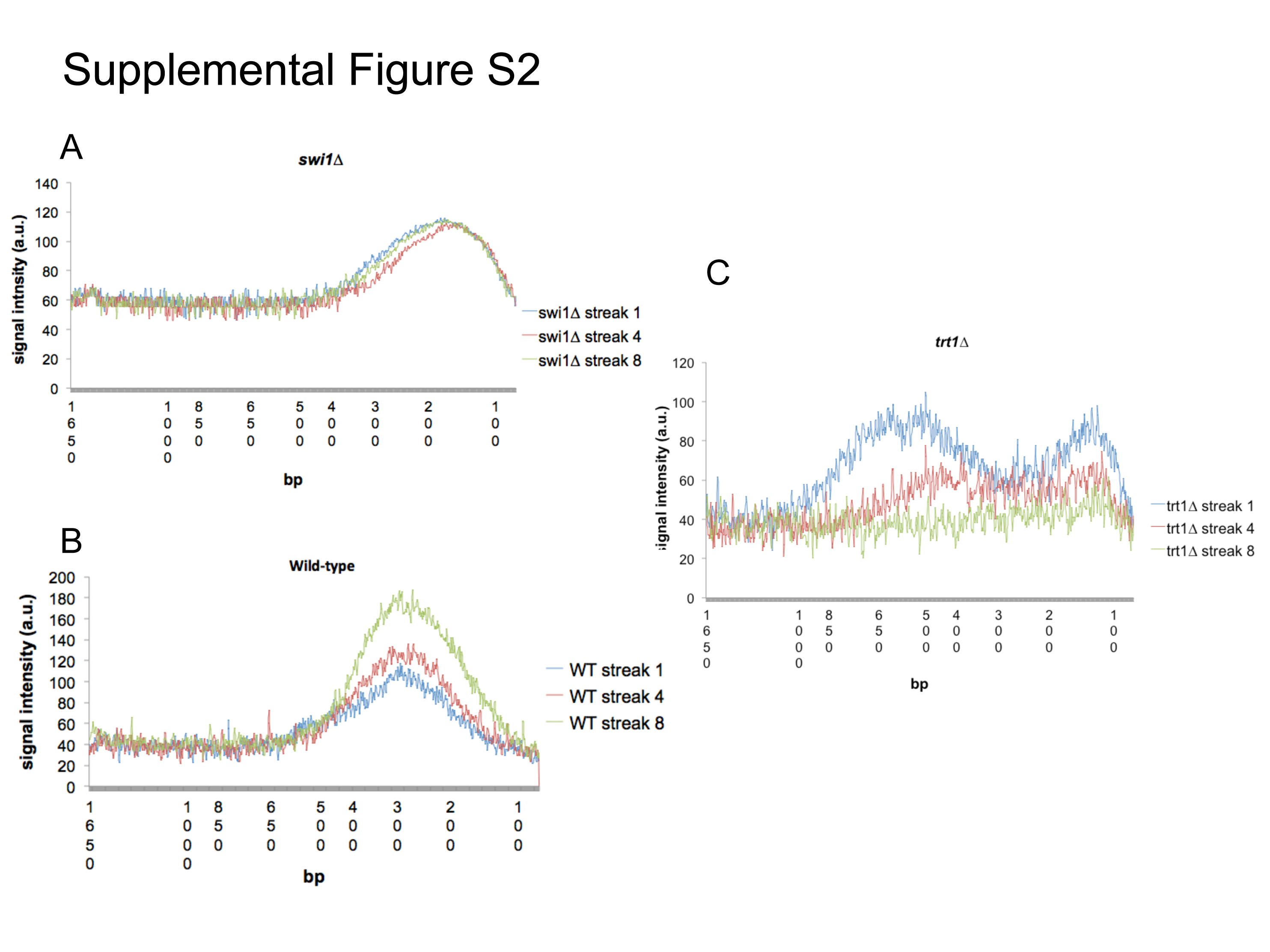

Supplement: S2 Fig — (A-C) Quantification of signal intensity of representative Southern blots of ApaI digested telomere fragments from wild-type (A), swi1Δ (B) and trt1Δ (C). Each image shows data of telomere lengths from DNA obtained after 1, 4, or 8 restreaks. Telomere fragments were detected using a telomere-specific probe as described in Fig 2B. Signal intensity quantification was performed with ImageJ software. (TIF) [file pgen.1005943.s002.tif]

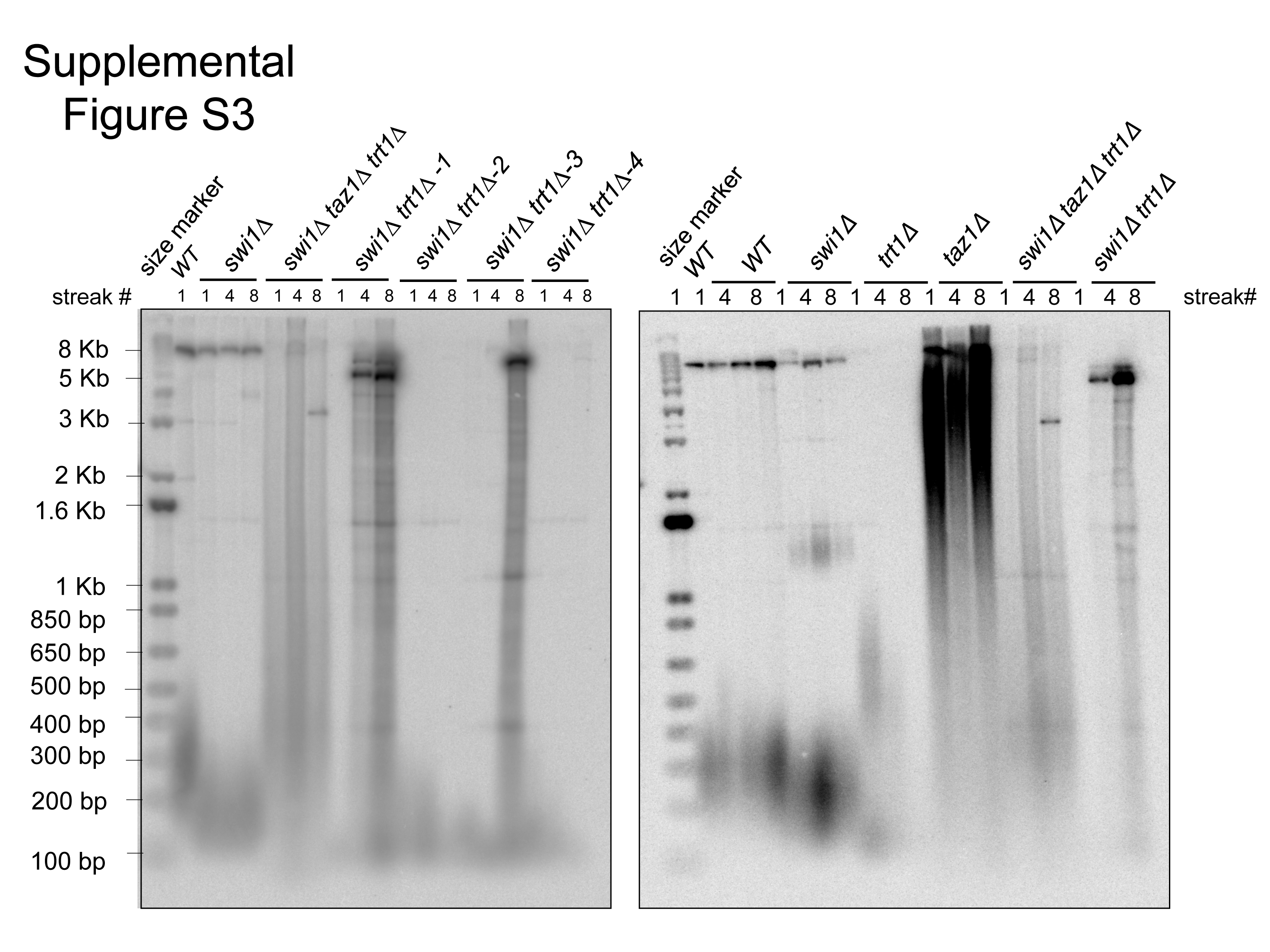

Supplement: S3 Fig — Southern blot analysis of telomere fragments from the indicated mutants. Strains were obtained by tetrad dissection. Genomic DNA was prepared after 1, 4, or 8 restreaks after the indicated strains were generated. ApaI-telomere fragments were detected using a telomere-specific probe as described in Fig 2B. A representative result is shown. (TIF) [file pgen.1005943.s003.tif]
